# Clinical treatment interventions in personal recovery stories of patients with severe mental illness: a qualitative study

**DOI:** 10.1007/s00127-025-02872-w

**Published:** 2025-03-11

**Authors:** Robin Michael Van Eck, Auke Jelsma, Jelle Blondeel, Kimriek de Wilde-Schutten, Jannick Vincent Rutger Zondervan, Thijs Jan Burger, Astrid Vellinga, Mariken Beatrijs de Koning, Frederike Schirmbeck, Sylvia Gerritsen, Martijn Kikkert, Lieuwe de Haan

**Affiliations:** 1 Department of Psychiatry, Amsterdam UMC, Location University of Amsterdam, Meibergdreef 9, Amsterdam, 1105 AZ The Netherlands; 2 Arkin, Institute for Mental Health, Amsterdam, The Netherlands; 3 VU University, Amsterdam, The Netherlands; 4 Department of Public Mental Health, Medical Faculty Mannheim, Central Institute of Mental Health, Heidelberg University, Mannheim, Germany; 5 Department of Ethics, Law and Humanities, Amsterdam UMC, Amsterdam, The Netherlands

**Keywords:** Personal recovery, Clinical recovery, Severe mental illness (SMI), Schizophrenia, Psychosis, Side-effects

## Abstract

**Purpose:**

In quantitative research, small to medium associations were found between clinical and personal recovery in patients with severe mental illness (SMI). This finding may result from varying relationships between clinical and personal recovery depending on the individual patient. The aim of the current study was to explore the subjective experience of clinical treatment interventions in personal recovery stories of patients with severe mental illness.

**Methods:**

Semi-structured interviews were conducted with 26 patients with SMI receiving treatment of a Flexible Assertive Community Treatment team in Amsterdam, the Netherlands. Thematic analysis was used.

**Results:**

We found that most clinical treatment interventions can have both positive and negative impact on personal recovery: (1) receiving a *diagnosis* can lead to relief, but also to stigma, (2) *medication* has positive effects, but side-effects impair personal recovery, (3) *hospitalization* and (4) *coercive treatment* can be helpful, but can also impact the process of recovery negatively, (5) *psychological treatment* is experienced as beneficial.

**Conclusion:**

Mental healthcare practitioners’ awareness of patients’ diverging experiences regarding the impact of clinical treatment interventions on personal recovery is important to carry out recovery-supportive practice. Communicating a diagnosis with a hopeful narrative, developing personalized medication strategies and post-hospital reflection on the use of restraints are a good basis.

## Introduction

Since the beginning of the 1990’s, recovery-oriented practices and research into recovery have emerged in mental health care worldwide [3, 49]. Recovery is a broad term, which in general medicine tends to be defined as ‘return to a pre-existing state of health’. In the field of mental healthcare, though, recovery is typically divided into two types: clinical and personal recovery [5, 51]. Sometimes, functional or societal recovery are distinguished as well (Castelein, Timmerman, PHAMOUS investigators, van der Gaag, & Visser, [11] Chan, Mak, Chio, & Tong) [12].

Clinical recovery refers to remission of symptoms and sometimes also includes functional improvement, mostly based on clinical rating scales or interviews, such as the PANSS (Positive and Negative Syndrome Scale) over at least a 6 month period [1, 26]; Liberman, Kopelowicz, Ventura, & Gutkind [32]; Torgalsbøen [56]. It usually has been defined as a medical view and an outcome. Personal recovery has been based on accounts of patients overcoming their illness and refers to a subjective appraisal of recovery [18, 38]. It is often described as an active, individual, unique, and non-linear process, or as a struggle or journey with different stages (Andresen, Oades, & Caputi [2], Anthony [3], Spaniol, Wewiorski, Gagne, & Anthony) [53]. Important processes, derived from recovery stories, are summarized in the acronym ‘CHIME’: ‘connectedness’, ‘hope’, ‘identity’, ‘meaning’ and ‘empowerment’ [6]; Leamy, Bird, Le Boutillier, Williams, & Slade [29]. Although personal recovery is often seen as a process, for (quantitative) research purposes it also has been defined and measured as an outcome [50].

In meta-analyses, small to medium associations were found between clinical and personal recovery in patients with a schizophrenia spectrum disorder [30]; Van Eck, Burger, Vellinga, Schirmbeck, & De Haan [60]. This finding may result from varying relationships between clinical and personal recovery depending on the individual patient.

Clinical recovery is supported by clinical treatment. It is important to gain more insight in which clinical treatment interventions are perceived by patients as helping or hindering, as clinicians may use this knowledge to adapt their practices to stimulate patients’ process of recovery [43].

Earlier qualitative studies suggest that the opinions of patients about clinical treatment interventions are very divergent (Forchuk, Jewell, Tweedell, & Steinnagel [24], P. Leendertse, Hirzalla, van den Berg, Castelein, & Mulder [31], Piat, Sabetti, & Bloom) [44]. Most of these studies have been performed in patients with schizophrenia spectrum disorders, although in clinical practice also patients with other diagnoses receive treatment in community mental health teams. Besides, earlier qualitative studies have not focused on the impact of a broad spectrum of clinical interventions on the personal recovery of patients, but usually on one or two aspects, for instance medication [21] or hospitalization [58].

Therefore, the main objective of the current study is to explore the subjective experience of a range of clinical treatment interventions in personal recovery stories of patients with severe mental illness (SMI), both in a helping and hindering sense.

## Methods

### Study design

This qualitative approach is part of a larger mixed-methods study. Firstly, a quantitative approach was used to investigate the relationship between clinical and personal recovery cross sectionally and over time. The research was set in the treatment context of five Flexible Assertive Community Treatment teams (F-ACT) of Mentrum, part of Arkin Institute for Mental Health, in the western part of Amsterdam, the Netherlands. F-ACT is a Dutch version of Assertive Community Treatment (ACT), aimed exclusively at patients with Severe Mental Illness (SMI) (Nugter, Engelsbel, Bähler, Keet, & van Veldhuizen [42]). SMI has different definitions worldwide, but we used the following: patients with prolonged psychiatric illness and need for long-term treatment (i.e. more than 2 years), who experience severe social, occupational, or school dysfunctioning, regardless of a specific diagnosis [20]; Ruggeri, Leese, Thornicroft, Bisoffi, & Tansella [47]. F-ACT teams offer a combination of ACT and individual case management, working with an outreaching method in the community. The F of F-ACT stands for a team that is flexible to temporarily intensify treatment to ACT if this is needed (Westen, Boyle, & Kroon) [67].

Patients were recruited for this research project by posters and flyers in the waiting room and all mental health workers were requested to ask their patients if they would be willing to participate in this research project. Participants consented to participate, after receiving an information leaflet explaining the aim of the study and interview procedure. Questionnaires about clinical and personal recovery were administered at baseline and three years later to patients with SMI. The characteristics of this study were described in earlier published articles (Van Eck, Burger, Schenkelaars, et al., [59] Van Eck et al., [61]. The original sample consisted of 105 patients. Of these, 90 gave permission to be contacted again for a follow-up interview. All participants met the inclusion criteria of having a SMI, being 18 years or older and being able to understand the Dutch language.

### Sampling strategy

Participants from the previously conducted quantitative study were approached again, now by a researcher, through telephone or e-mail correspondence, to participate in a qualitative interview. We used purposive sampling to select participants. We based the selection on the scores of patients on the quantitative measures of clinical and personal recovery, for instance a combination of high clinical recovery and low personal recovery, or low clinical recovery and low personal recovery, to ensure the most diverse views on the impact of clinical interventions on personal recovery. In other words, we aimed to explore a maximum variation in experiences, rather than representativeness for the total study population.

8 participants refused to participate on second thought with multiple reasons, for instance, no time, or not willing to talk for 30 minutes or more (which was longer than the time needed for the previously administered quantitative questionnaires). In total, 26 patients with a severe mental disorder (SMI) were interviewed.

### Data collection

The interviews took place between March 2017 and August 2019. A trained researcher (A.J., J.B., K.W., J.Z.) conducted the interviews face-to-face at the local treatment facility, or, if requested by the participant, at their own home. 3 interviewers were medical students (A.J., J.B., J.Z.) and K.W. had a background in health and life sciences. Interviews took approximately 30–90 min (mean: 56 min). Interviews were audio-recorded, for which participants gave consent before the interview.

For the semi-structured interviews, a topic list was used that started with questions that are considered appropriate to discover one’s personal recovery story because of their open, yet personal character: what has happened, what are strengths and weaknesses and what are future hopes or goals [64]. Following that, respondents were queried about helping and hindering factors in their recovery process. A substantial part of the interview time was spent on establishing rapport and giving the respondents space to tell their recovery story in their own way. Getting a global picture of the overall recovery story first was considered necessary to enable the interviewer to ask in more depth about the impact of clinical interventions on recovery. If clinical treatment was not spontaneously mentioned by respondents, participants were further prompted about the influence of specific interventions on their personal recovery: Receiving a diagnosis, Medication, Hospitalization, Coercive treatment, Psychological treatment. The two researchers that conceptualized the study (R.V.E. and A.V.), both psychiatrists, based the choice for asking about these specific interventions on their own clinical experience and medical training and on the existing literature on this subject, as mentioned in the introduction. We felt that these categories of treatment are applied in clinical practice from a mainly ‘medical’ point of view in line with a goal of clinical remission/recovery. The interview guide was not pilot tested, but the opening questions of the recovery-oriented interview have been used before in research [10].

### Data analysis

The interviews were transcribed verbatim with all identifiers removed and uploaded to the MAXQDA Qualitative Data Analysis Software package to facilitate analysis. Thematic analysis enables the generation of new insights derived from the data [9]. Exploring the data on how clinical treatment interventions impact personal recovery, we openly and inductively coded the experiences in the interviews, staying attentive towards potential other themes of clinical treatment apart from the above mentioned ones (diagnosis, medication, hospitalization, coercion, psychological treatment). The pre-defined categories that were explicitly asked for during the interviews, were used as a deductive framework for the results. The subthemes were inductively established. The coding of the data into the categories was done by the researcher who had also conducted the interview. 5 interviews were analyzed and coded by A.J., 5 by J.B, 6 by K.W. and 10 by J.Z. After a set of interviews was analyzed, R.V.E. and A.V. separately read all the interviews, discussed them with the interviewer and sought for agreement about the coding. They also judged if there was still new relevant knowledge obtained from the interviews. After the discussion, the final categorization was done. After 26 interviews views started to repeat, so it was concluded that there was data saturation.

### Ethical aspects

All participants provided informed consent. The study protocol was reviewed by the ethical committee of the Vrije Universiteit Medical Centre (FWA00017598, reference 2015.350), and was granted an exemption from approval based on the fact that participants in the study were not subject to procedures or interventions, or required to follow rules of behavior.

## Results

Of the 26 participants, 19 were male, 7 female. Ages ranged between 33 and 67. 18 participants had a schizophrenia spectrum disorder, 8 an affective or personality disorder.

We found that most clinical treatment interventions can have both positive and negative impact on personal recovery. Besides the categories defined during the design of the study, no additional overarching clinical themes were identified in the narratives.

**Fig. 1:**
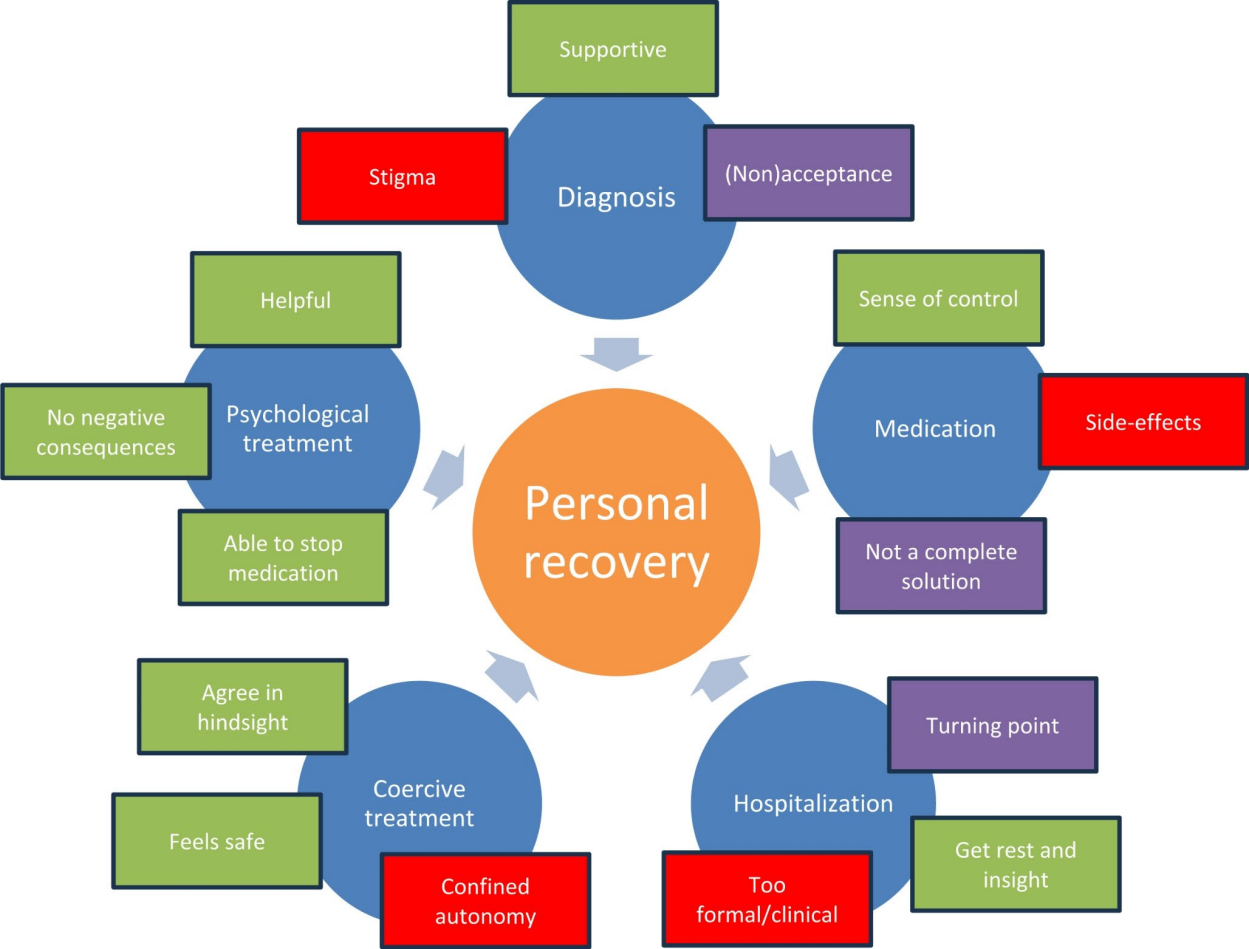
Graphic representation of the results, showing themes (in blue) and positive (in green), negative (in red) and combined subthemes (in purple) of clinical interventions impacting personal recovery

The results are graphically presented in Fig. 1.

The views of the participants on the clinical interventions are described in more detail below, divided into positive and negative experiences/subthemes:

1. Receiving a diagnosis.

*Positive experiences*

A substantial part of the respondents experienced receiving a diagnosis as supportive.

It helped a respondent refraining from self-blame for his problems:

*R: “During the first ten years it was all my fault: I didn’t have a job*, *I was going crazy*, *that was my fault as well*, *and I couldn’t handle anything. So*, *yeah*, *when you hear you’re schizophrenic*, *and that it’s just something in your head*, *in your blood*, *in your chemistry*, *then I’m not really to blame for all of it.” ~ P007*.

It also helped to accept illness and move on:

*R: “You have to accept it*, *yes. And if you can accept it*, *you can move on.” ~ P002*.

*Negative experiences*

Receiving a diagnosis was perceived as an obstruction in the way to recovery by several participants. The following respondent describes that having a psychiatric diagnosis is not as accepted as a physical diagnosis.

R: *“Sounds nice*, *‘psychiatric diagnosis’*, *but it means that you will have a disadvantage compared to physical disorders. Physical disorders are accepted*, *but mental disorders not.” ~ P040*.

Due to the diagnosis, participants experienced (self-)stigma and underestimation of possibilities to have contact with others. Respondent P053 felt judged by his brother, while P014 experienced self-stigma.

*R: “[My brother]*, *he really labels you. The label of psychiatry. He can’t view you as a normal person anymore.” ~ P053*.

*R: “Due to my illness*, *I developed a really bad self-image*, *and that’s also my weak point I think. I feel bad because of my illness*, *you know. Like some sort of stigma or whatever. Because of that I don’t feel completely full-fledged*, *as a person.” ~ P014*.

2. Medication.

*Positive experiences*

Many participants mentioned the importance of symptom reduction by the use of medication to achieve personal recovery goals. For some respondents the use of medication gave a sense of control, because they were not overwhelmed by symptoms anymore and could function more independently.

*I: “Can you tell me what makes you resilient against these kinds of symptoms?”*

*R: “I think*, *in the end*, *medication. It gives some control. When I take medication*, *I can discard them*, *the ideas. And sometimes when someone says something*, *I still think ‘He means this or that’*, *but then with medication… yeah*, *I can discard that idea*, *like ‘That’s not true.’” ~ P007*.

*R: “We’ve tried every medication*, *and finally clozapine*, *and that’s helped me to again continue my life independently.” ~ P014*.

*Negative experiences*

The majority of the respondents argued that side-effects can be a clear downside of medication.

*R: “Look*, *I found life horrible*, *it wasn’t doable with all those voices*, *severe psychosis. I happily take the medication because that was horrible. But yeah*, *that still leaves a big problem*, *I think. […]. I take medication that gives some people more energy*, *but not for me*, *I just fall asleep after taking it.” ~ P007*.

*‘* *Combined’ (both positive and negative) experience*

Also, it was stated by some that if medication does not work sufficiently, it is possible to live with ongoing symptoms. So, medication is not a complete solution.

*I: "Does the medication help you?"*

*R: "Well*, *my thoughts are a bit more ‘closed’ in a way [by the medication]. […] But on the other hand*, *they [the voices] enriched my life. Because these voices are negative and that helped me to become very positive [respondent smiles]" ~ P006*.

3. Hospitalization.

Most respondents had experienced admission to a psychiatric hospital at some point during their treatment. For many respondents, the moment of the first admission could be seen as some kind of ‘turning point’ in recovery, both positively and negatively.

*Positive experiences*

*R: “[…] it [admission] does of course give you insight. All of a sudden you realize you really are ill.” ~ P053*.

Most respondents argued that the admission was, in hindsight, necessary. They described that the admission had its positive effects, like enabling them to get rest in a stressful period, as well as insight in themselves and the acceptance of treatment that they needed.

*R: “On the day of the admission*, *you have a clean bed and a meal. And nothing had to be done. It was actually a liberation for me. It was absolutely not a bad experience. […] I really calmed down there. We were close to the sea and I often went biking*, *going into nature. So no*, *I have never experienced my admissions as bad.” ~ P067*.

*Negative experiences*

Nevertheless, some respondents also mentioned downsides of admission. One participant felt that the support of care professionals during the admission was not actively focused on recovery:

*R: “In the clinic the staff is nice*, *but also very distant; a little formal. I wasn’t very happy about it. Recovery is something you need to do by yourself there. They take care of the environment*, *but recovery is your own business.” ~ P031*.

4. Coercive treatment.

*Positive experiences*

Several participants called, in retrospect, coercive measures supportive. For example, forced admission was sometimes viewed as a protection from society or a safe environment:

R: “*It [forced admission] was very pleasant*, *because it felt safe. The door was locked and perhaps that was to protect the society against the ‘crazy’*, *but to me it felt like I was being protected from the society. I completely came to rest on that closed department… it is not dangerous [the closed department]*, *I found it a very safe environment.” ~ P031*.

Some acknowledged that they were not able to give informed consent and that they were glad in hindsight that the decision for forced admission was made by someone else.

*R: “Apparently*, *I was so confused that I could not have stayed at home. It [coercive admission] had to happen. I understand that it could not have been executed differently. If everyone says so*, *then who am I? I find it fantastic that one is helped like this*, *and the admissions went really well. I felt not ready to have any direction in the treatment*, *I was too confused*, *I still needed some direction.” ~ P058*.

*Negative experiences*

In contrast, by some participants coercive treatment with medication and forced hospital admission were perceived as having a negative impact on their lives. They felt their autonomy was confined and that they had not much to say about their treatment.

*R: “They told me: “you have to take this [medication]*, *otherwise we will admit you.’’*, *which is something I really want to prevent*, *for this seems the worst thing possible*, *as I already had to say so little about my own life. This would take away my last freedom*, *the last part of the little freedom that I had. […] It gave me a feeling that I had very little to say.” ~ P090*.

5. Psychological treatment.

Psychological interventions were, if applicable, viewed as helpful, and no negative consequences on their recovery process were mentioned by the respondents.

*“Yeah*, *that [EMDR] was a heavy therapy*, *but I was happy about it. I think it was a good therapy; I’m glad that it was offered to me. “ ~ P031*.

*R: “[…]*, *I had that psychologist*, *[…]. Those [the things I’ve learned] I won’t forget*, *so when I’m down in the dumps*, *I use those things I’ve learned.” ~ P066*.

One participant talked about the possibility of discontinuing medication because of doing psychological treatment.

*R: “[What I want to work on] are the compulsions […] with the help of the psychologist*, *so we’re discontinuing medication*, *I’m happy to say. We’re doing it verbally now*, *with training*, *or therapy.” ~ P004*.

## Discussion

The current study aimed to explore the subjective experience of clinical treatment interventions in personal recovery stories of patients with severe mental illness.

The role that specific clinical treatment interventions play for personal recovery appears to differ widely among SMI patients, ranging from very helpful to very hindering.

The following clinical treatment interventions were discussed in the personal recovery stories of the participants:

1. Receiving a diagnosis.

It was found that receiving a diagnosis can function as an important step towards personal recovery, while it can also hinder this process. A diagnosis may provide an explanation for complaints and can give access to treatment options. Also, it can endorse the idea that not the patient, but the illness is to blame for the symptoms. Conversely, receiving the diagnosis of a mental illness can lead to a negative self-image because of a stigma that can accompany the diagnosis. This negative influence on self-identity may be an extra obstacle for patients in their personal recovery process, which is in line with findings from previous studies (Link, Struening, Neese-Todd, Asmussen, & Phelan [33], Mashiach-Eizenberg, Hasson-Ohayon, Yanos, Lysaker, & Roe) [36]. The impact of a diagnosis of psychosis has been described before as a ‘means of access’ as well as a ‘cause of disempowerment’. It can help by ‘naming the problem’ and hinder by ‘labelling the person’. It can also be a ‘cause of social exclusion’ (Pitt, Kilbride, Welford, Nothard, & Morrison) [45]. Perceived and experienced stigma, also from mental health providers, predicts self-stigma (Dubreucq, Plasse, & Franck) [22].

It is important that health care workers are aware of the fact that a diagnosis can have these different consequences for patients. Furthermore, it is advisable to have a conversation about the impact of the diagnosis on the patient’s personal recovery process and to be alert about (self-)stigma [46]. ‘Stigma resistance’, defined as ‘one’s ability to deflect or challenge stigmatizing beliefs’ was found to be associated with advanced stages of personal recovery in serious mental illness patients [23]. Moreover, when clinicians give someone a diagnosis, a hopeful attitude and discussing the potential for recovery, are important.

2. Medication.

Medication can be of great help for patients’ personal recovery process by reducing symptoms. It can give a sense of control and can make patients able to function independently again. Regaining a sense of self has been described before [14]. Some participants mentioned positive experiences of symptoms, especially of psychosis. Schneider et al. found before that up to 54.4% of one-hundred twelve treatment-seeking patients with symptoms of psychosis reported that they would miss at least some aspects of positive symptoms if they should disappear [48]. Also, hearing voices can be experienced as intrusive, but also as providing guidance, depending on cultural context (Luhrmann, Padmavati, Tharoor, & Osei) [35]. Side-effects of medication, according to some participants, can interfere with the process of recovery. The subjective response to medication can differ substantially between patients [15].

Considering the subjective impact of psychotic symptoms, which may be positive and negative, it is important to decide to what extent one should pursue symptom reduction with antipsychotic medication. Whether positive symptoms are an important focus, could be assessed with the Subjective Perception of Positive Symptoms–Revised (SUPPOSY–R) [40]. Also, critical evaluation of positive effects and side-effects of medication is advisable, for instance with the Subjective Well-being Under Neuroleptic Treatment Scale (SWN) [41, 66]. Reducing or stopping medication has been mentioned as an intervention to help people feel less as a patient and to take away side-effects (Mathew, Nirmala, & Kommu) [37]. Also, dose-reduction/discontinuation strategies may help balance clinical and personal recovery [4]; Wunderink, Nieboer, Wiersma, Sytema, & Nienhuis) [68].

3. Hospitalization.

Admission to a psychiatric clinic was often seen as necessary and positive. Looking back, some also call it a ‘turning point’, making insight in themselves or acceptance of treatment possible. Not much research has been done into the impact of hospitalization on personal recovery. One study showed in thirty-four individuals with psychotic disorders with an average duration of the hospitalization of 81.9 days no significant changes in personal recovery, self-efficacy, and self-esteem, although clinical symptoms significantly improved [39].

Possibly, hospitalization is mostly focused on taking care of a crisis and reducing acute symptoms, which might not immediately has an impact on personal recovery. Also, the process of personal recovery can take multiple years and shows ups and downs [17].

It might be a good idea to evaluate an admission afterwards with a patient and family to decide to which extent a possible future admission should be included in the treatment plan to support personal recovery.

4. Coercive treatment.

The coercive interventions that were mentioned in the interviews were forced medication and hospitalization; seclusion was not mentioned. The results indicate that the perception of the use of these coercive measures tends to differ between patients. While some patients perceived coercive measures as negative, others did not share this view. As found in an earlier study, a sense of safety, agency, control and empowerment is important for coercive treatment to aid, rather than disrupt recovery (Wyder, Bland, & Crompton) [69]. Also, shame, self-contempt and stigma stress as a reaction to involuntary hospitalization showed poorer recovery after 2 years [70].

Due to the negative impact coercive measures can have, they should be used as a last resort and good communication with the patient is extremely important (Chieze, Hurst, Kaiser, & Sentissi [13], Tingleff, Bradley, Gildberg, Munksgaard, & Hounsgaard) [55]. If coercive measures are used, evaluation on the impact is essential with the patient and family, also to explore whether traumatic experiences need support or treatment.

5. Psychological treatment.

Psychological interventions are perceived by respondents to have a positive effect on personal recovery and no clear downsides were indicated. Patients consider it helpful to treat specific symptoms, such as depressive mood, anxiety, trauma related and psychotic symptoms with for instance Cognitive behavioral therapy (CBT) or Eye Movement Desensitization and Reprocessing (EMDR). Hancock and Perich already found in their review that recovery-focused interventions based on principles of CBT showed significant improvement in personal recovery of patients with bipolar disorder [25]. Participants of the current study also underscore the importance of paying attention to resilience factors, such as having a positive self-image/self-esteem. This is in line with earlier research, indicating that improving resilience contributes to personal recovery [7, 52, 57, 62].

The finding that no participants reported negatively about psychological interventions, may have to do with the fact that in order to start a psychotherapy there has to be some kind of mutual agreement about the problem to address and the relevance and purpose of the chosen psychological treatment. This differs from the other categories of interventions, because the latter can also be performed without consent of the specific patient (e.g. administer medication, hospitalization).

### Limitations and future research

A limitation of this study might be that patients who agreed and were able to participate in an interview of 30 to 90 min are relatively well-functioning and may have more positive views concerning their treatment.

In trying to make statements concerning the impact of factors on personal recovery there should be kept in mind the substantial differences between individual patients. We should be aware of potential over or under interpreting the meaning of a topic for someone’s personal recovery process.

In future research it is important to also include patients with even more severe symptoms and more impaired functioning, for instance in long-term inpatient mental health rehabilitation settings. There is generally an underrepresentation of this patient group, at least partly due to issues with informed consent [16]. Adding a category of ‘difficulties’ to the CHIME-framework of personal recovery, has been proposed to better understand the challenges of recovery [28]; Stuart, Tansey, & Quayle) [54]. Also, future qualitative research can focus on more specific questions, such as the consequences of stigmatizing attitudes of professionals (Defourny, van Sambeek, van de Bovenkamp, Scheepers, & Heerings) [19]. Investigating a larger span of narratives, e.g. from patients at risk of psychosis, or other under-researched mental health sub-populations can broaden the understanding of factors influencing personal recovery [8, 27, 34]. A ‘Psychiatry Story Bank’ is a way to collect diverging first person accounts (van Sambeek, Baart, Franssen, van Geelen, & Scheepers) [65]. During the performing of the current research we also considered to compare different subgroups of patients, based on scores on the quantitative recovery measures, but found it difficult to define cut-off scores and were afraid that this division would undermine the personal character of the narratives. However, future research can reconsider this. Efforts have been made before to identify such profiles of recovery (van Gestel-Timmermans, Brouwers, Bongers, van Assen, & van Nieuwenhuizen) [63]. Lastly, because personal recovery is a journey undertaken together with significant others, research into the collaboration on recovery within the triad of patient, family and professional is important [10].

## Conclusion

Patients with severe mental illness were interviewed to explore their subjective experience of clinical treatment interventions in their personal recovery stories.

Clinical treatment interventions have diverging impact on personal recovery: (1) receiving a *diagnosis* can lead to relief, but also to stigma, (2) *medication* has positive effects, but side-effects impair personal recovery, (3) *hospitalization* and (4) *coercive treatment* can be helpful, but can also impact the process of recovery negatively, (5) *psychological treatment* is experienced as beneficial.

Mental healthcare practitioners’ awareness of patients’ diverging experiences regarding the impact of clinical treatment interventions on personal recovery is important to carry out recovery-supportive practice that fits an individual patient’s needs. Highlights from the current study are: communicating a diagnosis with a hopeful narrative, developing personalized medication strategies and post-hospital reflection on the use of restraints.

## Data Availability

The data used and analyzed in this study are not publicly available, as these datasets contain information that could compromise participants’ privacy. The data supporting our findings is available from the corresponding author on reasonable request.
